# A Novel Noncoding RNA *dsr11* Involved in Heat Stress Tolerance in *Deinococcus radiodurans*

**DOI:** 10.3390/biom10010022

**Published:** 2019-12-23

**Authors:** Dong Xue, Yun Chen, Jiang Li, Jiahui Han, Zhengfu Zhou, Wei Zhang, Ming Chen, Min Lin, Marc Ongena, Jin Wang

**Affiliations:** 1 Biotechnology Research Institute, Chinese Academy of Agricultural Sciences, Beijing 100081, China; xue_dong_kevin@126.com (D.X.); chenyun0402ye@163.com (Y.C.); lijiangemail01@126.com (J.L.); 13121257599@163.com (J.H.); zhouzhengfu@caas.cn (Z.Z.); zhangwei01@caas.cn (W.Z.); chenming01@caas.cn (M.C.); linmin57@vip.163.com (M.L.); 2 Microbial Processes and Interactions (MiPI), TERRA Teaching and Research Centre, Gembloux Agro-Bio Tech, University of Liège, 5030 Gembloux, Belgium

**Keywords:** *Deinococcus radiodurans*, noncoding RNA, *dsr11*, heat stress, *trmE*, *dr_0651*

## Abstract

*Deinococcus radiodurans* is an extremely resistant bacteria that has evolved masterful strategies to enable survival under various environmental stress conditions. Heat stress is a major environmental stress factor that can cause denaturation of proteins, membrane disruption, and oxidative stress. Previous studies have examined the mechanisms of the heat stress response by analyzing changes in protein levels; however, little is known about the role of small noncoding RNAs (ncRNAs), which are known to play important regulatory functions in bacteria during various environmental stress response. The ncRNA *dsr11* of *D. radiodurans* was previously identified by RNA-seq and Northern blot. In this study, we showed that the transcription level of *dsr11* was up-regulated 4.2-fold under heat stress by qRT-PCR analysis. Heat tolerance assay showed that deleting *dsr11* significantly inhibited the viability under high temperature conditions. To assess the influence of *dsr11* on the *D. radiodurans* transcriptome, 157 genes were found differentially expressed in the knock-out mutant by RNA-seq experiment. Combining RNA-seq and in silico analysis, we found that *trmE* (tRNA modification GTPase) and *dr_0651* (arginase) were likely to be the direct targets of *dsr11*. Further microscale thermophoresis results demonstrated that *dsr11* can directly bind to the mRNA of *trmE* and *dr_0651*. Our results indicated that *dsr11* can enhance the tolerance to heat stress of *D. radiodurans* by binding to *trmE* and *dr_0651* mRNA. Overall, these results extend our understanding of ncRNA regulation and provide new insights into the heat stress response in *D. radiodurans*.

## 1. Introduction

*Deinococcus radiodurans* (*D. radiodurans*) is a Gram-positive, pink-pigmented, high G + C bacterium that belongs to the Deinococcus–Thermus phylum. It was isolated from gamma-radiated canned meat and is best known for its extraordinary resistance to ionizing irradiation [1,2,3]. However, previous studies also reported that this bacterium presents a rapid response and adaptation to a wide variety of extreme environment stresses, such as ultraviolet irradiation, desiccation, hydrogen peroxide, and temperature [4,5,6,7,8]. Due to its powerful DNA repair ability and extreme stress resistance, this bacterium has become a model for studying bacterial tolerance mechanisms under various environmental stress conditions. To adapt to rapid changes of environmental conditions, many genes associated with stress tolerance have been identified in *D. radiodurans* [9]. Although much effort has been carried out to elucidate the molecular mechanisms conferring high resistance capability in response to various environment in *D. radiodurans*, the complex post-transcriptional regulation and gene expression still requires deeper investigation.

Small noncoding RNAs (ncRNAs) are ubiquitously found in bacteria (50–500 nt), which have been divided into two groups (*cis*-encoded ncRNAs and *trans*-encoded ncRNAs) based on the location of their targets [10,11]. They usually function by repressing or activating gene expression via base-pairing with target mRNAs to modulate translational activation and mRNA stabilization [12]. Bacterial ncRNAs have been reported to act as vital regulator in response to various environment stresses, such as, virulence, pH, oxidative, antibiotic, and temperature [13,14,15,16,17]. Thanks to the development of biocomputational and experimental methods, a large number of ncRNAs have been found in prokaryotes [18]. In extremophilic bacteria and archaea such as *Methanococcus jannaschii*, *Bacillus halodurans*, and *Haloferax volcanii*, a large amount of ncRNA was found [19,20,21]. In particular, many of these ncRNAs are involved in the regulation of various stresses. For example, hundreds of differentially expressed ncRNAs in response to oxidative stress were identified in *H. volcanii* [22]. In *H. volcanii*, sRNA132 is important for rapid adaptation to phosphate starvation [23]. Recently, Tsai et al. [24] identified 41 ncRNA candidates in *D. radiodurans* by the genome-wide RNA sequencing approach but the functional roles of these ncRNAs are still poorly understood.

This study aimed at providing new insights into the environmental adaption of *D. radiodurans*. We characterized *dsr11* as a novel ncRNA but conserved among Deinococci and which is highly induced upon exposure to high temperature. We combined various experimental and in silico approaches in order to identify the potential targets of *dsr11* and the potential regulation pathway leading to heat tolerance in that bacterium.

## 2. Materials and Methods

### 2.1. Strain and Growth Conditions

*D. radiodurans* was obtained from the China General Microbiological Culture Collection Center (CGMCC 1.633, Beijing, China). *D. radiodurans* and derivatives were routinely cultured in TGY broth (1% tryptone, 0.5% yeast extract, and 0.1% glucose) or on TGY plates supplemented with agar (1.5%) at 30 °C. When required, ampicillin and kanamycin were added to final concentrations of 50 and 20 µg/mL, respectively.

### 2.2. RNA Extraction

Total cells from *D. radiodurans* were prepared using TRIzol reagent (Invitrogen, Thermo Fisher, California, USA) with Lysing Matrix Tubes (MP Bio, California, USA), and total cellular RNA was extracted with the PureLink RNA Mini Kit (Invitrogen, Thermo Fisher, California, USA) following the manufacturer’s instructions. RNA purity was assessed using absorbance readings (260 nm/280 nm) with a NanoDrop^®^ spectrophotometer (Thermo Fisher, California, USA).

### 2.3. Construction of Gene Deletion Mutant Strain

Mutant strain lacking *dsr11* was constructed by fusion PCR recombination of a kanamycin resistance cassette into the genome as previously described [25]. Briefly, fusion PCR products for *dsr11* deletion was constructed in two steps. In the first step, a pair of specific premiers were used to generate fragment complementary to the kanamycin-resistance gene from the plasmid pKatAPH3 (920 bp) and the upstream (414 bp) and downstream regions (417 bp) of *dsr11* sequences using the appropriate primer pairs (Supplementary Table S1). In the second step, the upstream, kanamycin-resistance gene, and downstream fragments were annealed at their overlapping regions and PCR amplified as a single fragment using the outer primers (1751 bp). The resulting PCR fragment was directly transformed into *D. radiodurans*. Colonies resistant to kanamycin (20 μg/mL) were selected, and these mutants were subsequently verified by PCR and DNA sequencing and named Δ*Dsr11*.

### 2.4. Quantitative Real-Time PCR (qRT-PCR)

cDNA synthesis was conducted by using a PrimeScript^TM^RT reagent kit with gDNA Eraser (TaKaRa, Kusatsu, Japan) as described in the manufacturer’s protocol. Subsequently, qRT-PCR was performed using ChamQ SYBR qPCR Master Mix (Vazyme Biotech Co., Ltd., Nanjing, China) on a 7500 Fast Real-time PCR System (Applied Biosystems, California, USA). The primers are listed in Supplementary Table S1. The 16S rRNA gene was used as the endogenous reference control to normalize differences in total RNA quantity, and relative gene expression was quantified by the 2^−ΔΔCT^ method. Three biological replicates for each condition were conducted.

### 2.5. Bacterial Growth Curve and Heat Stress Tolerance Assays

*D. radiodurans* wild-type (WT) and mutant (Δ*Dsr11*) strains were grown in shacked (220 rpm) TGY cultures in triplicate at 30 °C. The OD_600_ of each sample was measured every 4 h. WT and ∆*Dsr11* were cultured in TGY broth with appropriate antibiotics to OD_600_ = 2 at 30 °C and were then shifting to 48 °C for 4 h. Subsequently, 100 μL of the cell suspension was aliquoted into 900 μL of PBS, after which 10-fold serial dilutions were made for all strains, and 8 µL of each dilution was spotted onto TGY agar plates. These plates were incubated at 30 °C for 3 days before colony growth was observed and calculated. All assays were performed in triplicate.

### 2.6. RNA-seq and Data Analysis

WT and Δ*Dsr11* were cultured in TGY broth with appropriate antibiotics to OD_600_ = 2 at 30 °C. Then, the cells were harvested by centrifugation at 12,000 × *g* for 3 min and stored at −80 °C for RNA extraction. For each strain, total RNA was extracted from at least three independent biological replicates as described in RNA isolation section. A total of 1 μg of high-quality RNA per sample was used as the input material for library preparation. Sequencing libraries were generated using a VAHTS Total RNA-seq Library Prep Kit for Illumina^®^ (Vazyme, NR603) following the manufacturer’s recommendations. Then the well-prepared library was sequenced using the Illumina HiSeq X Ten platform with a 150-bp paired-end module. Raw reads were filtered by removing reads containing adapter, poly-N and low-quality reads for subsequent analysis. The resulting sequences were then mapped onto the *D. radiodurans* reference genome (NC_001263.1, NC_001264.1, NC_000958.1, and NC_000959.1) using TopHat (v2.1.1) [26]. The mapped reads of each sample were assembled using Cufflinks (v2.2.1) [27] with a reference-based approach. Cuffdiff (v2.2.1) [27] provides statistical routines for determining differential expression in a digital transcript or gene expression dataset using a model based on a negative binomial distribution. Genes with corrected *p* values less than 0.05 and absolute log_2_ values (Fold changes) >1 were considered significant differentially expressed genes (DEGs). All samples were sequenced three times. Gene ontology (GO) enrichment analysis of the DEGs was performed with the Perl module (GO::TermFinder) [28]. GO terms with a corrected *p* value less than 0.05 were considered to be significantly enriched among the DEGs. The Kyoto Encyclopedia of Gene and Genomes (KEGG) is a major public database containing manually drawn pathway maps representing knowledge of molecular interactions and reaction networks. R functions (phyper and q-value) were used to test for the statistical enrichment of the DEGs among the KEGG pathways. KEGG pathways with corrected *p*-values less than 0.05 were considered to be significantly enriched for the DEGs.

### 2.7. Bioinformatics Analysis

The secondary structure prediction performed with RNAalifold [29] based on the lowest folding energy. The sequence conservation of *dsr11* by using BLASTN. All sequenced bacterial genomes were compared with parameter values set to the default, and sequences with a nucleotide identity >60% and coverage value >80% considered as being conserved. The multi-sequence alignment of *dsr11* homologues performed with Clustal X2. The phylogenetic tree was constructed from the aligned sequences using Maximum Composite likelihood model with the UPGMA method, bootstrapped 1000 times via MEGA 7.0 software.

The target genes of *dsr11* were predicted by using Web-based program TargetRNA2 [30]. The hybridization between the ncRNA transcript sequence and the sequence comprising 75 nucleotides (nt) upstream until 75 nt downstream of the start codon of each annotated gene was screened in the genome of *D. radiodurans*. To consider an interaction as positive, we used the corresponding *p* < 0.05 and synonym energy less than −8 kcal/mol were taken as the threshold. Finally, the common predictions of TargetRNA2 and RNA-seq analysis confirmed as the target candidates.

### 2.8. Microscale Thermophoresis (MST) Analysis

MST technique were performed to quantify the interactions between ncRNAs and the target mRNAs in vitro [31,32,33]. The 5′FAM-labeled mRNAs of the target genes (*trmE* and *dr_0651*) were synthesized by the BGI company (BGI, Beijing, China). The ssRNAs oligonucleotides containing wild-type (*wt*) or mutated (*mut*) base-pairing regions of *dsr11* were synthesized by the BGI company (BGI, Beijing, China), as listed in Supplementary Table S2. The *wt* and *mut dsr11* probe molecules were labeled with 5′FAM. Four microliters of sample containing 200 nM labeled mRNA and increasing concentrations of a non-labeled competitor (from 18.3 nM to 600 mM) were loaded on standard treated glass capillaries (Monolith NT.115 Series Capillaries, Cat#MO-K002). Thermophoresis was carried out using a Monolith NT.115 instrument (NanoTemper Technologies, Munich, Germany) at 25 °C in diethylpyrocarbonate water with 40% excitation power and medium MST-Power. The dissociation constants (K_d_) were calculated as previously described [34]. Data analyses were performed using MO. Affinity Analysis software (NanoTemper Technologies, Munich, Germany).

## 3. Results and Discussion

### 3.1. Characterization of the Novel ncRNA dsr11

**Figure 1:**
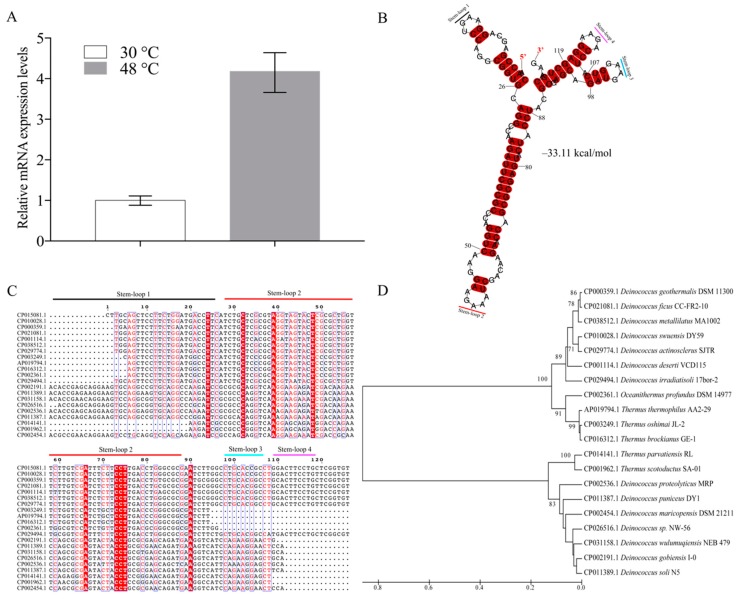
Secondary structure, multi-sequence alignment, and phylogenetic analysis of *dsr11*. (**A**) The expression of *dsr11* under heat stress in *D. radiodurans* wild-type (WT) by qRT-PCR. (**B**) *dsr11* consisted of four stem-loop structures with free energy of −33.11 kcal/mol, and the stem-loops were numbered. (**C**) The multi-sequence alignment was performed using Clustal X2 and then manually refined. The sequences were divided into four clusters. The sequences of stem-loop 2 were highly similar to other homologs. (**D**) The guide tree reflects the similarity relations between the homologous sequences in the form of hierarchical clustering. It is constructed by the UPGMA method by MEGA 7.0.

In a previous study, 41 ncRNAs were reported in *D. radiodurans* under normal conditions [24]. Here we further functionally analyzed these ncRNAs under different abiotic stresses (date not shown). Interestingly, we found a novel ncRNA named *dsr11*, which was 4.2-fold up-regulated upon growth at 48 °C (Figure 1A). This ncRNA was thus selected for further investigation during the heat stress response. Since the expression of *dsr11* was enhanced under heat stress, we hypothesized that *dsr11* might positively regulate heat tolerance in *D. radiodurans*.

According to the previous results of RNA-seq and deep RACE, the sequence of *dsr11* was 126 nt long. In general, ncRNAs form secondary structures, which contribute to their stability [10]. The secondary structure of *dsr11* was predicted by RNAalifold [29], and the four stem-loop structures with a free energy −33.11 kcal/mol may facilitate the high stability of *dsr11* (Figure 1B). It has been reported that ncRNAs can have conserved sequences and structures in homologs [35]. As shown in Figure 1C, *dsr11* is conserved among several *Deinococcus* species, such as *D. wulumuqiensis*, *D. gobiensis*, *D. geothermalis*, and *D. actinosclerus*, and it is also conserved in *Thermus* species (*T. thermophilus*, *T. brockianus*, and *T. scotoductus*) and *Oceanithermus profundus*. These *Thermus* species were reported to live at high temperatures, and *Oceanithermus profundus* was isolated from a deep-sea hydrothermal vent [36]. We hypothesize that *dsr11* may play an important role for these species in their environment. The multi-sequence alignment of *dsr11* against representative genomes of other microbes indicated that the sequences of stem-loop 2 had high similarity to other homologs, while similarity was clearly lower for stem-loop 1, 3, and 4 (Figure 1C). The transcript sequences were divided into four clusters by the sequence of stem loop 1, 3, and 4, which was in good agreement with the phylogenetic tree, as shown in Figure 1D. Although the sequences of ncRNAs were similar, they may retain different functions in various species due to different target binding regions [35]. The sequence and the stem-loop structures can be divided into four clusters probably related to different functions.

### 3.2. dsr11 is Required for Heat Tolerance of D. radiodurans

**Figure 2:**
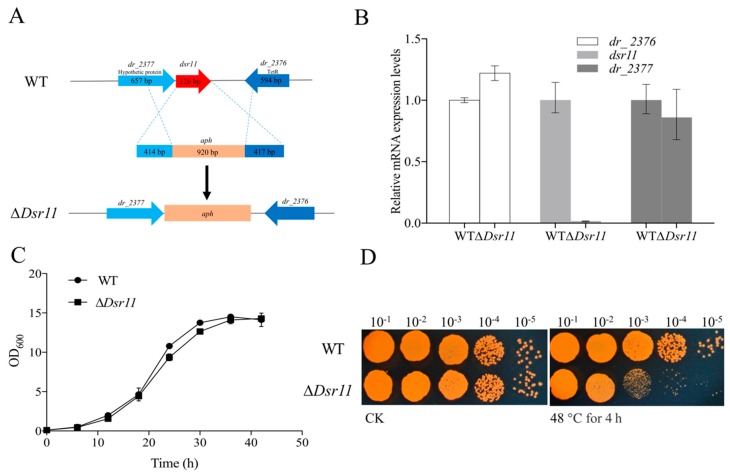
(**A**) The genomic location of *dsr11* and construction of the *dsr11* mutant. The *dsr11* is shaded in red. Schematic representation of the mutant generated by replacing the *dsr11* region with the kanamycin resistance gene *aph*. (**B**) Relative expression of *dsr11*, the upstream gene (*dr_2376*), and the downstream gene (*dr_2377*) in ∆*Dsr11* compared to the WT under normal conditions. The relative levels of the transcripts are presented as the mean values ± standard deviation, calculated from three sets of independent experiments and normalized to levels in the WT strain. (**C**) Growth curves in TGY broth of WT and ∆*Dsr11*. The error bars represent the calculated standard deviation of the measurements of three biological replicates. (**D**) Viability of *dsr11* knockout cells after exposure to 48 °C. CK, untreated culture control. All experiments were performed three times.

*dsr11* was located in the intergenic region between *dr_2376* (encoding a TetR family transcriptional regulator) and *dr_2377* (encoding a hypothetical protein) (Figure 2A). To further investigate the influence of *dsr11* on *D. radiodurans*, the knockout gene of *dsr11* was constructed successfully and named ∆*Dsr11* (Figure 2A). It was important to verify whether the deletion had a polar effect on the flanking genes; thus, we checked the expression by qRT-PCR of the flanking genes (*dr_2376* and *dr_2377*) and *dsr11* in WT and ∆*Dsr11*. As shown in Figure 2B, a significant difference of expression was observed only in the case of *dsr11* (expectedly expression could not be detected in the mutant), while there was no difference of *dr_2376* and *dr_2377* between WT and ∆*Dsr11*. The effect of *dsr11* on *D. radiodurans* growth at optimum growth temperature was then examined and there was no significant difference between the WT and ∆*Dsr11* (Figure 2C).

To determine the role of *dsr11* in *D. radiodurans* under heat stress, the WT and ∆*Dsr11* strains were exposed to high temperature conditions at 48 °C for 4 h. As shown in Figure 2D, the TGY plate assays showed that the mutant strain ∆*Dsr11* was more susceptible than the WT strain upon growth at 48 °C, confirming that *dsr11* serves an important role in the adaptation to heat stress.

### 3.3. RNA-seq Analysis Sheds New Light on the Mechanisms of dsr11-Mediated Heat Tolerance

**Figure 3:**
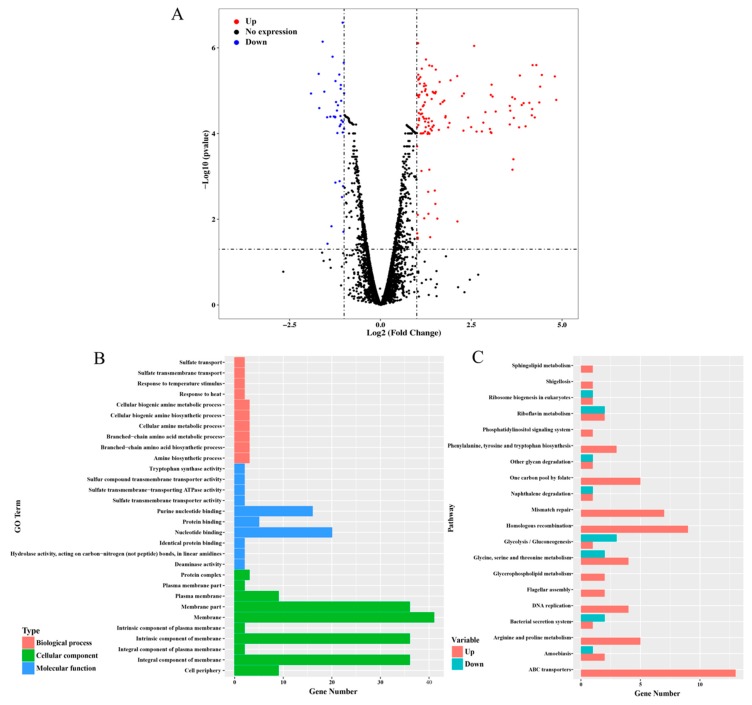
RNA-seq analysis between WT and ∆*Dsr11*. (**A**) Volcano map of the differential expression genes ∆*Dsr11* versus WT. Each point represents a unigene. The red and blue points indicate significant changes in the absolute value of log_2_ (fold change) ≥ 1 and FDR ≤ 0.05, respectively; i.e., the red points indicate up-regulated unigenes, and the blue points indicate down-regulated unigenes in the two groups, with the differential expression levels presented along the *X*-axis. The black points indicate nonsignificant, differentially expressed unigenes (**B**) GO terms with significant enrichment analysis of differentially expressed genes in ∆*Dsr11* compared to WT. (**C**) Top 20 KEGG biological pathway classification histograms for annotated unigenes.

ncRNA are usually involved in post-transcriptional regulation in various environment stresses. Thus, to expand the panel of how *dsr11* is involved in heat stress tolerance, and to have a better view of the effect of *dsr11* activity on the global transcription, an RNA sequencing transcriptome analysis was performed comparing ∆*Dsr11* to the WT strain. We observed 157 genes (Supplementary Table S3 and the most representative genes listed in Table 1) differentially expressed in the *dsr11* deletion mutant compared to the WT strain, including 121 genes that were up-regulated and 36 genes that were down-regulated (Figure 3A). Among the up-regulated and down-regulated genes in the *dsr11* deletion mutant we identified genes involved in the heat stress response (*csp*, *dnaJ*, *grpE*, *clpB*, and *hsp20*). The genome of *D. radiodurans* contains 3079 protein-coding genes [3] and forty-one ncRNAs were also identified [24]. Deletion of *dsr11* altered the expression of about 5.1% of all genes. These results indicated that *dsr11* directly or indirectly regulates these factors.

The function of the DEGs was determined by GO enrichment analysis, which were assigned as “membrane”, “membrane part”, “nucleotide binding”, “purine nucleotide binding”, “response to temperature stimulus”, and “response to heat” (Figure 3A). Knockout of the *dsr11* affects the expression of genes involved in cell membranes. Previous reports have shown that heat stress can lead to cell membrane disruption in *D. radiodurans* [37]. We hypothesized that the loss of *dsr11* may lead to changes in cell membrane function, thus affecting heat stress tolerance. In addition, both nucleotide binding and purine nucleotide binding are affected, which may also lead to sensitivity to heat stress. Through KEGG analysis, different pathways were significantly affected by *dsr11* knockout, including mismatch repair, homologous recombination, DNA replication, ABC transporters, and arginine and proline metabolism (Figure 3B). This result suggests that genes involved in these pathways may interact with *dsr11* to regulate the heat tolerance of *D. radiodurans*, which still requires further validation.

### 3.4. Identification of the dsr11 Targets

In bacteria, ncRNAs are commonly known to regulate target mRNAs through sequence-specific base pairing [11]. Base pairing resulting in target activation can involve ncRNA interactions with the 5′ untranslated region (UTR), the coding sequence, or the 3′ UTR of the target mRNAs, in many respects, functionally analogous to eukaryotic miRNAs [11]. The region of potential base pairing between ncRNAs and target mRNAs typically encompasses 10–25 nt [38]. The majority of these ncRNAs frequently repress or activate stress response pathways required for adaptation to changing environments. Bacterial ncRNAs activate translation or enhance stability of one or more mRNA targets [10,39]. Therefore, the identification and validation of putative ncRNA targets is essential to understand the roles of these regulators in bacteria. We used the webservers TargetRNA2 [30] to predict the potential targets of *dsr11*, and a total of 35 targets were identified according to the parameters used (Supplementary Table S4).

**Figure 4:**
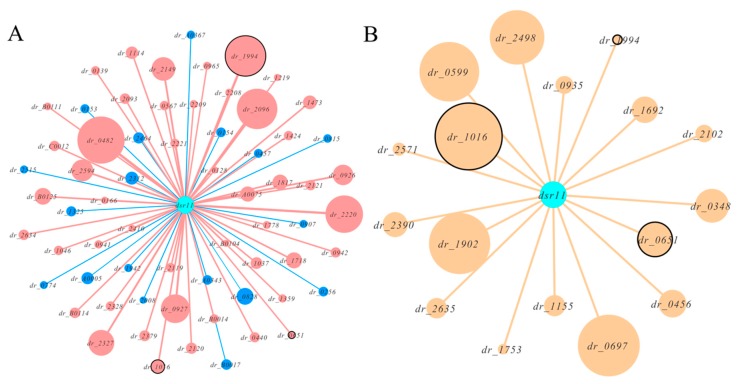
Network plots of *dsr11* and target interactions by RNA-seq and in silico prediction. All hypothetical genes were eliminated. (**A**)The effect of *dsr11* knockout on gene expression by RNA-seq. Red nodes represent up-regulated genes and blue nodes represents down-regulated genes. The size of the node represents the expression. The common genes as in silico predicted are marked with black circles. (**B**) The predicted targets of *dsr11* by TargetRNA2. The size of the node represents the binding strength based on the thermodynamic energy of hybridization. The common genes as RNA-seq analysis are marked with black circles.

**Figure 5:**
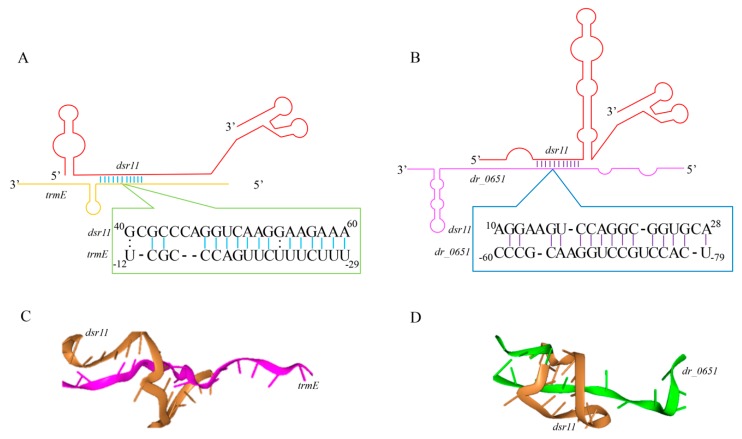
Predicted interaction sites of *dsr11* and target genes. (**A**,**B**) Schematization of the interaction between *dsr11* and two target mRNAs based on TargetRNA2. (**C**,**D**) The tri-dimensional interaction model was predicted by HNADOCK [46].

We combined this in silico target prediction and the results from RNA-seq analysis and identified three genes (two known genes and a hypothetical gene) common in these two approaches (Figure 4). We selected the two known genes *dr_1016* (*trmE*, coding for tRNA modification GTPase) and *dr_0651* (coding for arginase) as the candidates for experimental validation. The binding sites between these two predicted targets and *dsr11* were analyzed (Figure 5). *trmE* was characterized in *E. coil*, *Pseudomonas syringae*, and *Thermotoga maritima*, and is involved in various stress responses [40,41,42]. *trmE* plays an essential role in translation, signal transduction, protein synthesis, and tRNA modification [43]. Arginase is an important enzyme in the urea cycle, which is responsible for catalysis of L-arginine in L-ornithine and urea [44]. The best characterized arginase is from *Helicobacter pylori*, in which it helps the cells tolerate acid stress [45]. We hypothesize that *dsr11* enhances heat tolerance by imperfect base-pairing to *trmE* and *dr_0651* 5’ UTR, thereby affecting the transcription and translation of these two genes.

**Figure 6:**
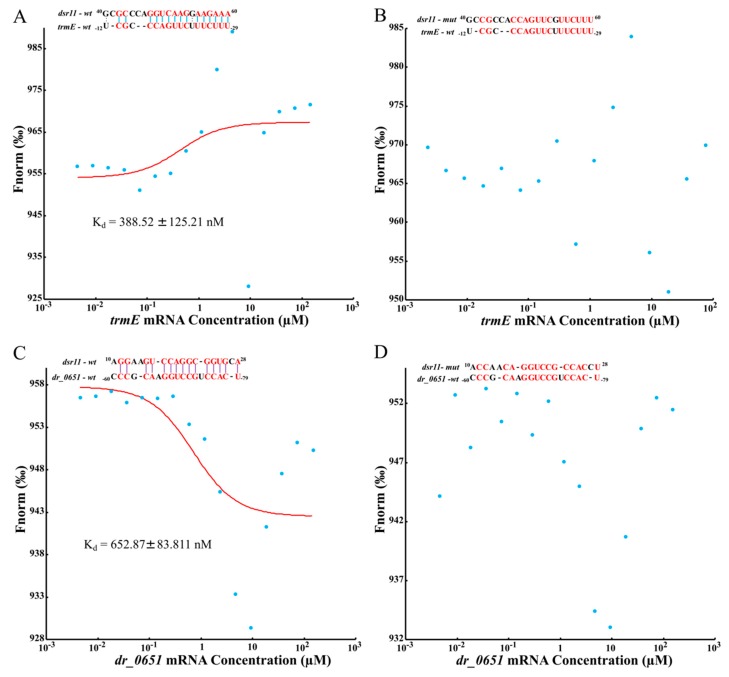
Determination of the binding affinity of *dsr11* to target mRNAs by microscale thermophoresis (MST). Red bases mean complementary bases in the binding sequence, the red curve is the fitted combination curve, and the Kd (dissociation equilibrium constant) value is the binding constant of *dsr11* and their targets. (**A**,**C**) are the binding of *trmE* mRNA and *dr_0651* mRNA, respectively, with *dsr11-wt*. (**B**,**D**) are the binding of *trmE* mRNA and *dr_0651* mRNA, respectively, with *dsr11-mut*.

To further validate whether *dsr11* binds directly to the target genes, MST was applied to identify the binding strength between fluorescently labeled *dsr11-wt* or *dsr11-mut* probes and the non-labeled competitor molecule on target genes *trmE* and *dr_0651* mRNA. Results indicated that *dsr11-wt* binds *trmE* and *dr_0651* mRNA at low micromolar concentrations in the titrant, exhibiting a dissociation constant (K_d_) of 388.52 ± 125.21 nM and 652.87 ± 83.811 nM, respectively, which suggests a relatively strong interaction (Figure 6A,C). In contrast, the mutant derivative (*dsr11-mut*) harboring substitutions in all complementary bases displayed a complete defect in binding to *trmE* and *dr_0651* mRNA (Figure 6B,D), suggesting that *dsr11* binds to the target genes through different bases. The binding assay showed that *dsr11* can regulate multiple genes in different cellular pathways, indicating that *dsr11* is required for adaptation to extreme environments and probably has specific regulatory functions in *D. radiodurans*.

## 4. Conclusions

In this study, we identified and characterized a novel ncRNA *dsr11* that contributes to heat tolerance in *D. radiodurans*. It is conserved among the class Deinococci with four stem-loop structures and is homologous to thermophilic bacteria. Phenotypic characterization of the mutant strain ∆*Dsr11* revealed that it was more susceptible than the WT to heat stress. Furthermore, RNA-seq analysis indicated that *dsr11* deletion affected the transcriptional expression of genes related to heat stress response, metabolism, and mismatch repair in *D. radiodurans*. The combination of in silico prediction and RNA-seq analysis allowed us to identify the tRNA modification GTPase gene *trmE* and arginase gene *dr_0651* as direct targets of *dsr11*. Our findings elucidate a potential regulation mechanism of ncRNA *dsr11*. However, the other possible mechanisms of *dsr11* interaction with target mRNA involved in heat stress tolerance remain to be fully understood.

## Figures and Tables

**Table 1:** Selection of the most representative genes differentially expressed in *D. radiodurans dsr11* deletion mutant with Log_2_ (FC) >1 or Log_2_ (FC) < 1.

Gene Name	Log_2_ (FC) *	*p* Value	Description
Down-regulated in ∆*Dsr11* strain			
*dr_0828*	−1.9173	1.17×10^−5^	Isocitrate lyase
*dr_2464*	−1.38547	4.06×10^−5^	Peptidyl-prolyl cis-trans isomerase, FKBP-type
*dr_1325*	−1.2897	4.02×10^−5^	Endopeptidase-related protein
*dr_0815*	−1.1394	4.23×10^−6^	Transcriptional regulator, GntR family
*dr_A0367*	−1.12989	1.3×10^−4^	UDP-galactopyranose mutase
dr_0907†	−1.08722	1.73×10^−5^	Cold shock protein, CSD family
Up-regulated in *∆Dsr11* strain			
*dr_0128*†	1.00099	1.29×10^−5^	GrpE protein
*dr_0166*	1.03439	4.26×10^−6^	Acyl-CoA-binding protein
*dr_0651*	1.10117	3.56×10^−5^	Arginase
*dr_1359*	1.13075	3.08×10^−6^	Outer membrane protein
*dr_0139*	1.1902	7.89×10^−6^	GTP-binding protein HflX
*dr_1424*†	1.23589	9.26×10^−6^	DnaJ protein
*dr_1046*†	1.24258	7.63×10^−6^	ATP-dependent Clp protease, ATP-binding subunit ClpB
*dr_2328*	1.25488	4.53×10^−6^	Sensor histidine kinase
*dr_0440*	1.30399	9.42×10^−5^	Holliday junction resolvase
*dr_0942*	1.31442	7.5×10^−4^	Tryptophan synthase, alpha subunit
*dr_1114*†	1.51777	1.07×10^−5^	Heat shock protein, HSP20 family
*dr_1473*	1.64409	2.04×10^−5^	Phage shock protein A
*dr_1016*	1.70629	1.85×10^−5^	trmE; tRNA modification GTPase TrmE
*dr_1994*	4.24133	4.23×10^−5^	Hypothetical protein

* Log_2_ (FC) = log_2_ of fold change calculated as ratio between gene expression of WT vs. ∆*Dsr11*. † Genes involved in heat stress tolerance regulation.

## Supplementary Materials

The following are available online at https://www.mdpi.com/2218-273X/10/1/22/s1. Table S1: Primers used in this study; Table S2: Synthesized ssRNA oligonucleotide derivatives for MST; Table S3: List of genes differentially expressed in the *dsr11* deletion mutant compared to the WT strain; Table S4: The possible targets of *dsr11* predicted by TargetRNA2.

## References

[1] Daly M.J. (2009). A new perspective on radiation resistance based on *Deinococcus radiodurans*. Nat. Rev. Microbiol..

[2] Omelchenko M.V., Wolf Y.I., Gaidamakova E.K., Matrosova V.Y., Vasilenko A., Zhai M., Daly M.J., Koonin E.V., Makarova K.S. (2005). Comparative genomics of *Thermus thermophilus* and *Deinococcus radiodurans*: Divergent routes of adaptation to thermophily and radiation resistance. BMC Evol. Biol..

[3] Hua X., Hua Y. (2016). Improved complete genome sequence of the extremely radioresistant bacterium *Deinococcus radiodurans* R1 obtained using PacBio singlemolecule sequencing. Genome Announc..

[4] Meyer L., Coste G., Sommer S., Oberto J., Confalonieri F., Servant P., Pasternak C. (2018). DdrI, a cAMP receptor protein family member, acts as a major regulator for adaptation of *Deinococcus radiodurans* to various stresses. J. Bacteriol..

[5] Slade D., Radman M. (2011). Oxidative stress resistance in *Deinococcus radiodurans*. Microbiol. Mol. Biol. Rev..

[6] Airo A., Chan S.L., Martinez Z., Platt M.O., Trent J.D. (2004). Heat shock and cold shock in *Deinococcus radiodurans*. Cell Biochem. Biophys..

[7] Patel B.A., Moreau M., Widom J., Chen H., Yin L., Hua Y., Crane B.R. (2009). Endogenous nitric oxide regulates the recovery of the radiation-resistant bacterium *Deinococcus radiodurans* from exposure to UV light. Proc. Natl. Acad. Sci. USA.

[8] Mattimore V., Battista J.R. (1996). Radioresistance of *Deinococcus radiodurans*: Functions necessary to survive ionizing radiation are also necessary to survive prolonged desiccation. J. Bacteriol..

[9] Blasius M., Sommer S., Hübscher U. (2008). *Deinococcus radiodurans*: What belongs to the survival kit?. Crit. Rev. Biochem. Mol. Biol..

[10] Gottesman S. (2005). Micros for microbes: Non-coding regulatory RNAs in bacteria. Trends Genet..

[11] Waters L.S., Storz G. (2009). Regulatory RNAs in bacteria. Cell.

[12] Storz G., Vogel J., Wassarman K.M. (2011). Regulation by small RNAs in bacteria: Expanding frontiers. Mol. Cell.

[13] Mann B., van Opijnen T., Wang J., Obert C., Wang Y.D., Carter R., McGoldrick D.J., Ridout G., Camilli A., Tuomanen E.I. (2012). Control of virulence by small RNAs in *Streptococcus pneumoniae*. PLoS Pathog..

[14] Opdyke J.A., Kang J.G., Storz G. (2004). GadY, a small-RNA regulator of acid response genes in *Escherichia coli*. J. Bacteriol..

[15] Zhang H., Zhan Y., Yan Y., Liu Y., Hu G., Wang S., Yang H., Qiu X., Liu Y., Li J. (2019). The *Pseudomonas stutzeri*-specific regulatory ncRNA, NfiS, targets the katB mRNA encoding a catalase essential for optimal oxidative resistance and nitrogenase activity. J. Bacteriol..

[16] Lybecker M.C., Samuels D.S. (2007). Temperature-induced regulation of RpoS by a small RNA in *Borrelia burgdorferi*. Mol. Microbiol..

[17] Eyraud A., Tattevin P., Chabelskaya S., Felden B. (2014). A small RNA controls a protein regulator involved in antibiotic resistance in *Staphylococcus aureus*. Nucleic Acids Res..

[18] Sharma C.M., Vogel J. (2009). Experimental approaches for the discovery and characterization of regulatory small RNA. Curr. Opin. Microbiol..

[19] Puerta-Fernandez E., Barrick J.E., Roth A., Breaker R.R. (2006). Identification of a large noncoding RNA in extremophilic eubacteria. Proc. Natl. Acad. Sci. USA.

[20] Klein R.J., Misulovin Z., Eddy S.R. (2002). Noncoding RNA genes identified in AT-rich hyperthermophiles. Proc. Natl. Acad. Sci. USA.

[21] Gelsinger D.R., Diruggiero J. (2018). The non-coding regulatory RNA revolution in archaea. Genes.

[22] Gelsinger D.R., DiRuggiero J. (2018). Transcriptional landscape and regulatory roles of small noncoding RNAs in the oxidative stress response of the haloarchaeon *Haloferax volcanii*. J. Bacteriol..

[23] Kliemt J., Jaschinski K., Soppa J. (2019). A haloarchaeal small regulatory RNA (sRNA) is essential for rapid adaptation to phosphate starvation conditions. Front. Microbiol..

[24] Tsai C.H., Liao R., Chou B., Contreras L.M. (2015). Transcriptional analysis of *Deinococcus radiodurans* reveals novel small RNAs that are differentially expressed under ionizing radiation. Appl. Environ. Microbiol..

[25] Sheng D., Gao G., Tian B., Xu Z., Zheng Z., Hua Y. (2005). RecX is involved in antioxidant mechanisms of the radioresistant bacterium *Deinococcus radiodurans*. FEMS Microbiol. Lett..

[26] Kim D., Pertea G., Trapnell C., Pimentel H., Kelley R., Salzberg S.L. (2013). TopHat2: Accurate alignment of transcriptomes in the presence of insertions, deletions and gene fusions. Genome Biol..

[27] Trapnell C., Roberts A., Goff L., Pertea G., Kim D., Kelley D.R., Pimentel H., Salzberg S.L., Rinn J.L., Pachter L. (2012). Differential gene and transcript expression analysis of RNA-seq experiments with TopHat and Cufflinks. Nat. Protoc..

[28] Boyle E.I., Weng S., Gollub J., Jin H., Botstein D., Cherry J.M., Sherlock G. (2004). GO::TermFinder-Open source software for accessing Gene Ontology information and finding significantly enriched Gene Ontology terms associated with a list of genes. Bioinformatics.

[29] Bernhart S.H., Hofacker I.L., Will S., Gruber A.R., Stadler P.F. (2008). RNAalifold: Improved consensus structure prediction for RNA alignments. BMC Bioinform..

[30] Kery M.B., Feldman M., Livny J., Tjaden B. (2014). TargetRNA2: Identifying targets of small regulatory RNAs in bacteria. Nucleic Acids Res..

[31] Beckert B., Kedrov A., Sohmen D., Kempf G., Wild K., Sinning I., Stahlberg H., Wilson D.N., Beckmann R. (2015). Translational arrest by a prokaryotic signal recognition particle is mediated by RNA interactions. Nat. Struct. Mol. Biol..

[32] Buddeweg A., Sharma K., Urlaub H., Schmitz R.A. (2018). sRNA41 affects ribosome binding sites within polycistronic mRNAs in *Methanosarcina mazei* Gö1. Mol. Microbiol..

[33] Jerabek-Willemsen M., André T., Wanner R., Roth H.M., Duhr S., Baaske P., Breitsprecher D. (2014). MicroScale Thermophoresis: Interaction analysis and beyond. J. Mol. Struct..

[34] Lippok S., Seidel S.A.I., Duhr S., Uhland K., Holthoff H.P., Jenne D., Braun D. (2012). Direct detection of antibody concentration and affinity in human serum using microscale thermophoresis. Anal. Chem..

[35] Peer A., Margalit H. (2011). Accessibility and evolutionary conservation mark bacterial small-RNA target-binding regions. J. Bacteriol..

[36] Miroshnichenko M.L., L’Haridon S., Jeanthon C., Antipov A.N., Kostrikina N.A., Tindall B.J., Schumann P., Spring S., Stackebrandt E., Bonch-Osmolovskaya E.A. (2003). *Oceanithermus profundus gen. nov., sp. nov.*, a thermophilic, microaerophilic, facultatively chemolithoheterotrophic bacterium from a deep-sea hydrothermal vent. Int. J. Syst. Evol. Microbiol..

[37] Bauermeister A., Hahn C., Rettberg P., Reitz G., Moeller R. (2012). Roles of DNA repair and membrane integrity in heat resistance of *Deinococcus radiodurans*. Arch. Microbiol..

[38] Kawamoto H., Koide Y., Morita T., Aiba H. (2006). Base-pairing requirement for RNA silencing by a bacterial small RNA and acceleration of duplex formation by Hfq. Mol. Microbiol..

[39] Prévost K., Salvail H., Desnoyers G., Jacques J.F., Phaneuf É., Massé E. (2007). The small RNA RyhB activates the translation of shiA mRNA encoding a permease of shikimate, a compound involved in siderophore synthesis. Mol. Microbiol..

[40] Yamanaka K., Hwang J., Inouye M. (2000). Characterization of GTPase activity of TrmE, a member of a novel GTPase superfamily, from Thermotoga maritima. J. Bacteriol..

[41] Singh A.K., Pindi P.K., Dube S., Sundareswaran V.R., Shivaji S. (2009). Importance of trmE for growth of the psychrophile *Pseudomonas syringae* at low temperatures. Appl. Environ. Microbiol..

[42] Gong S., Ma Z., Foster J.W. (2004). The Era-like GTPase TrmE conditionally activates gadE and glutamate-dependent acid resistance in *Escherichia coli*. Mol. Microbiol..

[43] Singh A.K., Shivaji S. (2010). A cold-active heat-labile tRNA modification GTPase from a psychrophilic bacterium *Pseudomonas syringae* (Lz4W). Res. Microbiol..

[44] Caldwell R.B., Toque H.A., Narayanan S.P., Caldwell R.W. (2015). Arginase: An old enzyme with new tricks. Trends Pharmacol. Sci..

[45] McGee D.J., Zabaleta J., Viator R.J., Testerman T.L., Ochoa A.C., Mendz G.L. (2004). Purification and characterization of *Helicobacter pylori* arginase, RocF: Unique features among the arginase superfamily. Eur. J. Biochem..

[46] He J., Wang J., Tao H., Xiao Y., Huang S.Y. (2019). HNADOCK: A nucleic acid docking server for modeling RNA/DNA-RNA/DNA 3D complex structures. Nucleic Acids Res..

