# Supplementary material for: A Novel Noncoding RNA dsr11 Involved in Heat Stress Tolerance in Deinococcus radiodurans
Source: Biomolecules. 2019 Dec 23;10(1):22. doi: 10.3390/biom10010022 (PMC7022480; doi:10.3390/biom10010022)
Supplement: Supplementary file 1 [file biomolecules-10-00022-s001.zip › supplementary files/Table S1, S2 and S4.docx]

Supplementary Material

## Supplementary Tables

**Supplementary Table S1** Premiers used in this study

| **Primers** | **Sequence (5’ – 3’)** | **Purpose** |
| --- | --- | --- |
| 16S | F: ATTCCTGGTGTAGCGGTG | qRT-PCR |
|  | R: CATCGTTTAGGGTGTGGAC |  |
| Dsr11 | F: GAGGACGCAGAAGAACAGC |  |
|  | R: GAGCAGTTCTCTTCAGACCTGAC |  |
| DR_2376 | F: GTTCGACGAGGCGCTGTTC |  |
|  | R: GGCTCGGCGGAAAGGTGCTTC |  |
| DR_2377 | F: GAATGGACCAGAGCCGCCTGATGAG |  |
|  | R: GTCTTTGCCGGTCGCGGCCTTGTAG |  |
| Dsr11-U | F: GCAACCTGATTCTGTCTGCCGTG | Dsr11 mutant construction |
|  | R: GCTCGGTCTCCATGCTCTGCCCCCACTTCGATAAC |  |
| Dsr11-M | F: GTGGGGGCAGAGCATGGAGACCGAGGGCCCTTG |  |
|  | R: GCAGTTCTCTTCTTAGAAAAACTCATCGAGCATC |  |
| Dsr11-D | F: CGAGTTTTTCTAAGAAGAGAACTGCTCAGGCGC |  |
|  | R: CGCGCAGCAGGTGTACGACTAC |  |

**Supplementary table S2** Synthesized ssRNA oligonucleotide derivatives for MST

| Name | Sequence (5’ – 3’) | Relevant characteristics |
| --- | --- | --- |
| dsr11-wt（5'FAM） | GCGCCCAGGUCAAGGAAGAAA | interaction with and trmE-wt |
| dsr11-wt（5'FAM） | AGGAAGUCCAGGCGGUGCA | interaction with and dr_0651-wt |
| dsr11-mut（5'FAM） | GCCGCCACCAGUUCGUUCUUU | Dismatch mutation, no interaction with trmE-wt |
| dsr11-mut（5'FAM） | ACCAACAGGUCCGCCACCU | Dismatch mutation, no interaction with dr0651-wt |
| dr0651-wt | UCACCUGCCUGGAACGCCC | interaction with dsr11-wt |
| trmE-wt | UUUCUUUCUUGACCCGC | interaction with dsr11-wt |

**Supplememtary Table S4** The possible targets of *dsr11* predicted by TargetRNA2

| **Rank** | **Gene ID** | **Description** | **Energy** | **Pvalue** |
| --- | --- | --- | --- | --- |
| 1 | DR_1576 | hypothetical protein | -17.09 | 0.000 |
| 2 | DR_2456 | hypothetical protein | -15.08 | 0.001 |
| 3 | DR_1016 (trmE) | tRNA modification GTPase TrmE | -14.99 | 0.001 |
| 4 | DR_0697 | v-type ATP synthase subunit E | -14.3 | 0.001 |
| 5 | DR_1902 | exodeoxyribonuclease V subunit RecD | -14.23 | 0.001 |
| 6 | DR_1312 | hypothetical protein | -14.18 | 0.001 |
| 7 | DR_0599 | aminoglycoside N3-acetyltransferase | -14.07 | 0.002 |
| 8 | DR_0520 | hypothetical protein | -14.03 | 0.002 |
| 9 | DR_1639 | hypothetical protein | -13.8 | 0.002 |
| 10 | DR_0818 | hypothetical protein | -13.55 | 0.002 |
| 11 | DR_2498 | GGDEF family protein | -13.51 | 0.003 |
| 12 | DR_1821 | hypothetical protein | -13.03 | 0.004 |
| 13 | DR_0552 | hypothetical protein | -12.5 | 0.005 |
| 14 | DR_0650 | hypothetical protein | -12.46 | 0.005 |
| 15 | DR_0651 | arginase | -11.49 | 0.010 |
| 16 | DR_0348 | cytochrome c-type biogenesis heme exporter protein C | -11.28 | 0.012 |
| 17 | DR_0895 | hypothetical protein | -11.18 | 0.012 |
| 18 | DR_1852 | hypothetical protein | -10.7 | 0.016 |
| 19 | DR_0456 | biopolymer transport protein | -10.61 | 0.017 |
| 20 | DR_2545 | hypothetical protein | -10.51 | 0.018 |
| 21 | DR_1692 | long-chain fatty acid--CoA ligase | -10.48 | 0.018 |
| 22 | DR_2390 | homoserine kinase | -10.32 | 0.020 |
| 23 | DR_1155 | malate synthase | -9.91 | 0.024 |
| 24 | DR_2635 | pyruvate kinase | -9.83 | 0.025 |
| 25 | DR_0935 | phosphatase | -9.55 | 0.029 |
| 26 | DR_2102 | plasmid stability protein | -9.51 | 0.030 |
| 27 | DR_1897 | hypothetical protein | -9.39 | 0.031 |
| 28 | DR_1529 | hypothetical protein | -9.26 | 0.033 |
| 29 | DR_0538 | hypothetical protein | -9.15 | 0.035 |
| 30 | DR_2571 (moaC) | molybdenum cofactor biosynthesis protein MoaC | -9.03 | 0.037 |
| 31 | DR_1587 | hypothetical protein | -8.79 | 0.041 |
| 32 | DR_1994 | hypothetical protein | -8.76 | 0.041 |
| 33 | DR_1753 | septum site-determining protein | -8.75 | 0.041 |
| 34 | DR_1688 | hypothetical protein | -8.64 | 0.043 |
| 35 | DR_1729 | hypothetical protein | -8.29 | 0.050 |
